# Presence of Enniatins and Beauvericin in Romanian Wheat Samples: From Raw Material to Products for Direct Human Consumption

**DOI:** 10.3390/toxins9060189

**Published:** 2017-06-12

**Authors:** Oana Stanciu, Cristina Juan, Doina Miere, Felicia Loghin, Jordi Mañes

**Affiliations:** 1 Department of Bromatology, Hygiene, Nutrition, Faculty of Pharmacy, “Iuliu Haţieganu” University of Medicine and Pharmacy, 6 Louis Pasteur, 400349 Cluj-Napoca, Romania; oana.stanciu@umfcluj.ro (O.S.); dmiere@umfcluj.ro (D.M.); 2 Laboratory of Food Chemistry and Toxicology, Faculty of Pharmacy, University of Valencia, Av. Vicent Andrés Estellés s/n, Burjassot, 46100 Valencia, Spain; jordi.manes@uv.es; 3 Department of Toxicology, Faculty of Pharmacy, “Iuliu Haţieganu” University of Medicine and Pharmacy, 6 Louis Pasteur, 400349 Cluj-Napoca, Romania; floghin@umfcluj.ro

**Keywords:** emerging mycotoxins, LC-MS/MS, cereals, organic, conventional, wheat products, estimated daily intake

## Abstract

In this study, a total of 244 wheat and wheat-based products collected from Romania were analyzed by liquid chromatography tandem mass spectrometry (LC-MS/MS) in order to evaluate the presence of four enniatins (ENs; i.e., ENA, ENA1, ENB, and ENB1) and beauvericin (BEA). For the wheat samples, the influence of agricultural practices was assessed, whereas the results for the wheat-based products were used to calculate the estimated daily intake of emerging mycotoxins through wheat consumption for the Romanian population. ENB presented the highest incidence (41% in wheat and 32% in wheat-based products), with its maximum levels of 815 μg kg^−1^ and 170 μg kg^−1^ in wheat and wheat-based products, respectively. The correlation between the concentrations of ENB and ENB1 in wheat grain samples and farm practices (organic or conventional) was confirmed statistically (*p* < 0.05). This is the first study that provides comprehensive information about the influence of agricultural practice on emerging *Fusarium* mycotoxin presence in Romanian wheat samples and the estimated daily intake of ENs and BEA present in wheat-based products for human consumption commercialized in Romania.

## 1. Introduction

Wheat (*Triticum aestivum* L.) is the main strategic crop worldwide, with recent data reporting a total area harvested of 224.7 million hectares, and an annual global production around 734 million metric tones [1]. In Romania, wheat has a special contribution in traditional agriculture, with 2.04 million hectares being used for wheat cultivation, with an annual production of about 7.85 million tons [2]. Moreover, the Romanian population registers a high wheat and wheat product consumption (133.09 kg/capita/year) [1].

Cereal grains are vulnerable to infections by a wide variety of plant pathogens. Filamentous fungi are a main safety concern due to the production of mycotoxins accumulated in grains as secondary metabolites [3]. The use of different agricultural practices, such as conventional or organic, could play an important role in the growth of various fungi and the biosynthesis of mycotoxins [4]. Production of organic wheat implies a management system that avoids the use of synthetic fertilizers, pesticides, herbicides, or genetically modified organisms. Due to the lack of synthetic fungicides in organic production, speculation has arisen about a possible higher contamination with different mycotoxins in this farming procedure in comparison with conventional practices. Most of the studies performed to clarify this hypothesis were based on trichothecene, fumonisin, or aflatoxin concentrations, and literature presents heterogeneous statistic results with no general conclusion established. Therefore the issue remains an open area for research [5].

Mycotoxins continue to attract worldwide attention because of their various toxic effects on human health, thus different maximum permitted levels (MLs) in foodstuffs and tolerable daily intakes (TDIs) for some mycotoxins such as aflatoxins (AFLAs), ochratoxin A (OTA), zearalenone (ZEA), deoxynivalenol (DON), fumonisins, and patulin have been set [6,7]. However, the emerging *Fusarium* mycotoxins including enniatins (ENs), beauvericin (BEA), fusaproliferin (FP), and moniliformin (MON) [8] are not legislated yet, despite their increasing detection influenced by both increasing frequency and sensitivity of the analysis methods [9].

ENs (ENA, ENA1, ENB, and ENB1) are biosynthesized especially by *Fusarium* sp. (*F. acuminatum*, *F. avenaceum*, *F. equiseti*, *F. langsethiae*, *F. lateritium*, *F. poae*, *F. sambucium*, *F. sporotrichioides*) [10], and BEA is produced mostly by *F. proliferatum*, *F. subglutinans*, *F. verticillioides*, or *F. oxysporum* [11], under the influence of several factors, such as geographic, topographic, climatic, biologic, or management related factors, additional to the preceding crop. The toxicity of these compounds is based on their ionophoric properties, disrupting normal concentrations of ions like K^+^ and Ca^2+^ across membranes [11,12]. ENs and BEA possess a wide range of potential properties: antimicrobial, anthelmintic, insecticidal, antifungal, herbicidal, phytotoxic, immunosuppressive, and cytotoxic [13,14]. Due to their similar chemical structures, ENs and BEA can present additive or synergistic cytotoxic effects, as has been demonstrated by in vitro studies [15,16].

In the literature, a special focus on type B ENs was observed, with ENB1 presenting the highest cytotoxicity on CHO-K1 cells and producing major disturbances on HepG2 cell cycle [13,17]. Also, the combination ENB1 + ENA1 is presented as the most cytotoxic combination of emerging mycotoxins on CHO-K1 cells, followed by ENA + ENB, ENA + ENA1 + ENB, and ENA + ENA1 + ENB1 [17]. This information has particular importance in the context of the high occurrence of ENB and ENB1, and their co-occurrence with other mycotoxins. Different studies have provided evidence that the incidence of ENB and ENB1 has reached high values in wheat and wheat products like flour, breakfast cereals, pasta or pizza, but, the influence of farming systems on emerging mycotoxins presence has been less studied and it is still ambiguous.

The occurrence and toxicity of ENs and BEA are under evaluation, and recently the European Food Safety Authority (EFSA) presented a scientific opinion about these mycotoxins [18]. Results of different studies on the occurrence of ENs and BEA in wheat grains or products from Finland, Norway, Germany, Sweden, The Netherlands, and several Mediterranean countries were presented, but no study from Romania was included in this report. Analyzing the present information about the emerging mycotoxins, EFSA had the following recommendations: the use of liquid chromatography tandem mass spectrometry (LC-MS/MS) methods to analyze ENs and BEA in food and feed, including prepared grain based products; monitoring the co-occurrence with other *Fusarium* toxins and the possible combined effects; and new research on in vitro and in vivo genotoxicity [18].

Due to the scarce information about emerging mycotoxin presence in Romanian wheat and its products, the aims of this work were: (i) to survey the levels of ENs and BEA in Romanian wheat and commercialized wheat-based products applying a LC-MS/MS method; (ii) to evaluate the differences regarding occurrence, co-occurrence, and concentration levels between organic and conventional crops; and (iii) to estimate the daily intake of emerging mycotoxins through wheat-based product consumption for the Romanian population. To the best of our knowledge, this is the first survey concerning the presence of emerging mycotoxins in wheat from Romania by agricultural practice and varieties and the first time when the estimated daily intake (EDI) through wheat-based product consumption in Romania has been calculated.

## 2. Results

### 2.1. Presence of Emerging Mycotoxins

#### 2.1.1. Wheat

A LC-MS/MS method was applied in order to evaluate the presence of ENA, ENA1, ENB, ENB1, and BEA in a total of 133 organic and conventional Romanian wheat samples. Sixty-one wheat samples (46%) presented detectable levels of ENs or BEA. Levels between limits of detection (LDs) and limits of quantification (LQs) were found in six situations: two for ENA, one for ENB, one for ENB1, and two for BEA. ENB was the most detected (55/133, 41%), followed by ENB1 (36/133, 27%), ENA1 (27/133, 20%), ENA (11/133, 8%), and BEA (3/133, 2%). Simultaneous contamination was observed in 28% of the samples analyzed (61% of the wheat positive samples were contaminated with two to four emerging mycotoxins). The means of only the positive samples (above LQs) were 65.8, 67.6, 135, and 116 μg kg^−1^ for ENA, ENA1, ENB, and ENB1, respectively. Only one wheat sample presented quantifiable levels of BEA (9.1 μg kg^−1^).

#### 2.1.2. Conventional versus Organic Wheat

**Figure 1:**
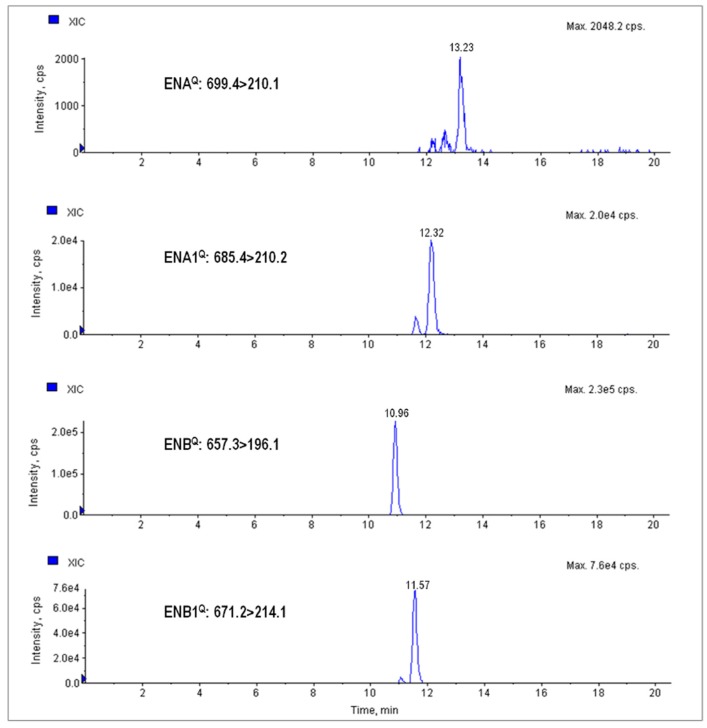
Selected reaction monitoring (SRM) chromatograms of a conventional wheat sample contaminated simultaneously with ENA (140 μg kg^−1^), ENA1 (356 μg kg^−1^), ENB (394 μg kg^−1^), and ENB1 (510 μg kg^−1^).

Concerning the organic wheat samples, 42 samples (70%) were contaminated with at least one emerging mycotoxin, which was more than the 19 conventional wheat samples (40%) that presented positive samples. As can be seen in Table 1, ENB was the most frequent mycotoxin in both types of wheat. The incidences of ENA, ENA1, ENB, and ENB1 were higher for the samples of organic wheat (19%, 30%, 70%, and 41%, respectively) than the corresponding values for conventional wheat (6%, 18%, 34%, and 24%, respectively). Mean values for the four ENs were higher for the organic samples (7.0, 23.7, 102, and 66.0 μg kg^−1^ for ENA, ENA1, ENB, and ENB1, respectively) (Table 1). Figure 1 presents the chromatogram of a conventional wheat sample contaminated with four ENs. On the other hand, detectable levels of BEA were found only in three conventional wheat samples at low concentrations, two of them being between the LD and LQ for BEA.

**Figure 2:**
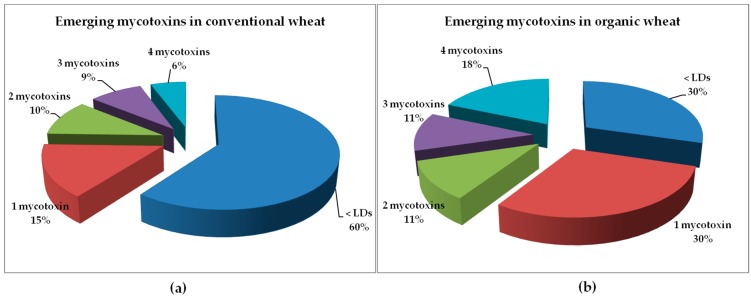
Mycotoxin frequency data for Romanian wheat: (**a**) conventional; (**b**) organic.

Emerging mycotoxin co-occurrence was found in 26 of 42 positive conventional wheat samples (62%), and 11 of 19 positive organic wheat samples (58%). For conventional wheat, the most frequent was the presence of two mycotoxins in the same sample, while for organic wheat the most frequent was the simultaneous contamination with four emerging mycotoxins. Detailed results for co-occurrence are presented in Figure 2 and Table 2. It must be remarked that ENB and ENB1, considered the most toxic ENs, were found together in all positive samples of organic wheat, and in 22 of 26 positive samples of conventional wheat (Table 2).

The results of one-way analysis of variance (ANOVA) revealed statistically significant differences (*p* < 0.05) between the two agricultural practices for ENB (*p* = 0.031) and ENB1 (*p* = 0.018) concentrations. Nevertheless, for ENA and ENA1, no statistically significant difference was observed, while for BEA, the test was not applied due to the low number of positive samples.

With respect to the results for conventional wheat by cultivars, from the 106 conventional wheat samples analyzed, 55 samples were classified, and were determined to belong to twenty-two varieties cultivated in Romania, and 51 samples belonged to other unclassified types of wheat. Final results showed that eleven varieties were negative for all emerging mycotoxins evaluated: Alcantara, Alex, Dropia, Felix, Hyfi, ITC-20, Lukulus, Miranda, Ponomicus, Solehio, and Urbanus. BEA was detected in three conventional wheat samples included in the group “Other types”, one being quantifiable (9.1 μg kg^−1^). Concerning the wheat varieties positive for ENs, the lowest levels (mean not exceeding 4 μg kg^−1^) were obtained for Arezzo, Boema, Glosa, and Kontrast varieties, while the highest means and levels were for Arieşan, Balaton, and Izvor varieties (Table 3).

#### 2.1.3. Wheat-Based Products

A total of 111 wheat-based products for direct human consumption (flour, pasta, breakfast cereals, and biscuits) commercialized in Romania were analyzed by LC-MS/MS to evaluate the presence of ENA, ENA1, ENB, ENB1, and BEA. Thirty-five wheat-based samples (32%) presented detectable levels of ENA1, ENB, EN1, and BEA. ENB was the most detected (35/111, 32%), followed by ENB1 (18/133, 16%), ENA1 (1/111, 1%), and BEA (1/111, 1%) (Table 4). Levels between LDs and LQs were found in eleven situations, as is presented in Table 4.

Quantifiable levels were found only for ENB and ENB1, ranging from 1.2 to 170 and from 2.2 to 44.8 μg kg^−1^, respectively. The averages of the positive samples (above LQs) were 17.5 μg kg^−1^ and 8.7 μg kg^−1^ for ENB and ENB1, respectively. The highest mean values of ENB and ENB1 (10.4 and 1.9 μg kg^−1^, respectively) and the highest levels for these mycotoxins (170 and 44.8 μg kg^−1^, respectively) were found in pasta, followed by flour, biscuits, and breakfast cereals. Simultaneous contamination was observed in 16% of the samples analyzed (51% of the positive samples were contaminated with two or three emerging mycotoxins). The most frequent situation was the contamination with two mycotoxins simultaneously (Table 2).

Regarding the organic samples of wheat-based products, the results showed that only one flour sample presented a detectable level of ENB (1.25 μg kg^−1^), and the other mycotoxins were not detected.

### 2.2. Dietary Exposure

To assess the risk of the Romanian population related to the exposure to emerging mycotoxins through the wheat product consumption, the EDIs were calculated at two different levels (Table 5).

At a low bound scenario (LB), the EDIs of the emerging mycotoxins analyzed ranged from 0 (BEA, ENA, and ENA1) to 25.8 ng/kg bw/day (ENB), whereas the EDIs at the upper bound scenario (UB) ranged between 5.3 (BEA) and 31.7 ng/kg bw/day (ENA). All EDI values calculated for ENs were lower than the hypothetic TDI proposed by the authors for the sum of ENs (1000 ng/kg bw/day) [19,20]. The total contribution of ENs to the hypothetic TDI for the Romanian population was 3.12% and 8.05% for LB and UB scenario, respectively (Table 5).

## 3. Discussion

In general, the presence of emerging mycotoxins in Romanian wheat samples found in our study (ENB presenting the highest incidence—41%, with its maximum of 815 μg kg^−1^) is in agreement with those of other European studies that reported ENB as the most frequent emerging mycotoxin in wheat. Alkadri et al. [21] found that 42% of the Italian wheat samples analyzed were contaminated with ENB at levels between 3.1 and 87.2 μg kg^−1^. On the other hand, recent studies presented higher values for occurrence and concentrations of emerging mycotoxins in wheat. For example, a study carried out by Juan et al. [22] reported that 78% of the wheat samples were positive for ENB, in concentrations ranging from 23 to 1826 μg kg^−1^. Similar results were presented by Bryła et al. [23] when evaluating mycotoxin occurrence in wheat samples from Poland; they found that 94% of the samples were contaminated with ENB at concentrations between 1 to 1981 μg kg^−1^.

One part of the present study was focused on the comparison between frequencies, mean, and maximum levels of emerging mycotoxins in conventional and organic cropping procedures used for wheat in Romania, and, to the best knowledge of the authors, represents the first study of this type. Differences in EN presence were observed, particularly for type B ENs, while for BEA no variations were noted. Higher values for incidences, mean levels, and number of mycotoxins found simultaneously were registered for organic wheat samples, but, interestingly, the maximum values found were for conventional samples. This could be explained by the multi-factorial influence in fungal growth and mycotoxin development based on plant substrate, topographic factors, weather parameters, or different management activities [4].

Relating to previous studies about emerging mycotoxin presence in wheat or wheat-based products by farm practice, until now, two published studies compared conventional and organic systems using only wheat-based products. Indeed, Jestoi et al. [24], studying the occurrence of sixteen *Fusarium* mycotoxins including six emerging mycotoxins (four ENs, as well as BEA and FP) in different conventional and organic grain-based products, observed that the highest concentrations of ENB (170 μg kg^−1^) and ENB1 (71 μg kg^−1^) were in conventional products. On the other hand, Serrano et al. [25], comparing organic and conventional pasta, found that 100% of the organic samples and 88% of the conventional samples were contaminated by at least one emerging mycotoxin. Moreover, the incidence percentages of ENA, ENB, ENB1, and BEA were higher in samples of organic pasta, while the concentration levels revealed a heterogeneous distribution: the mean level of ENA (7.3 μg kg^−1^) was higher for organic wheat pasta, the mean levels of ENB (12.8 μg kg^−1^) and ENB1 (18.8 μg kg^−1^) were higher for conventional wheat pasta, and, concerning BEA and ENA1, no significant differences were observed.

The literature presents similar comparisons for wheat or wheat derivatives produced organically or conventionally, using also the levels of other mycotoxins. A study analyzing ten trichothecenes and ZEA in 247 organic and 1377 conventional wheat samples distributed across the whole of the United Kingdom and over five harvest years, identified no significant differences in DON and ZEA concentrations between organic and conventional samples, while the incidence and concentration of positive samples for HT-2 and T-2 toxins were both significantly lower for the organic samples [26]. Bernhoft et al. [27] reported significantly lower *Fusarium* infestation and levels of DON, HT-2, and T-2 toxins in samples of organic cereals such as wheat, barley, and oats in comparison with the paired samples of conventional cereals cultivated in Norway, similar with the results of an Italian study [28] that observed a higher contamination by *Fusarium* spp. in conventional wheat in comparison to organic wheat. Regarding wheat products, organic and conventional wheat flour samples commercialized in Croatia in 2008 and 2009 were evaluated for OTA and ZEA presence and no statistical differences between organic and conventional products were observed [29]. The same statistical conclusion was also reached by a research group from Slovenia, even if the contamination rate with AFLAs, OTA, fumonisins, DON, ZEA, HT-2, and T-2 toxins was higher for the organic cereal products [30]. In addition, different authors [31] declared that crop management system is the weakest factor influencing the internal colonization of winter wheat kernels by *Fusarium* fungi.

Due to the high demand for organic foodstuffs and the occurrence of some mycotoxins in wheat, including emerging mycotoxins, research on wheat quality from different farming systems is relevant. Moreover, taking into account the differences in reporting, analytical methods and sensitivities obtained, statistical analyses applied, and agriculture particularities, it is recommended that continuous monitoring studies should be conducted in all types of wheat in an effort to reach final conclusions about best practices in order to inform policies.

The correlation between the presence of emerging mycotoxins in Romanian wheat and the wheat variety is highlighted for the first time in the present study. Detailed analysis for mycotoxin contamination by variety criterion was previously performed in Romania only for DON, when the highest levels of DON were found for Alex, Arieşan, and Exotic wheat varieties, while lower means and concentration levels were observed for wheat varieties such as Balaton, Boema, Glosa, and Ponomicus [32].

Concerning wheat crops in Romania, the most used varieties are: Boema (25% of wheat crops), Glosa (16.5%), and Dropia (16.1%). Boema wheat variety is resistant to Romanian winter conditions, scorching heat and drought; also, it is resistant to yellow rust, medium resistant to brown rust, and it presents better resistance to sprout damages than other varieties. Glosa variety is medium sensible to brown rust, and medium resistant to yellow rust, *Septoria* contamination, and wheat head fusariosis, while Dropia variety is known to be tolerant to scorching heat and drought, resistant to *Septoria* contamination, medium resistant to yellow and brown rust, and sensible to wheat head fusariosis. These varieties are recommended for Romanian hill and plain areas [32]. Knowing that Boema, Glosa, and Dropia are the most cultivated wheat varieties, and considering the low levels of emerging mycotoxins in our study, but also the previous study on DON, it can be accepted that safe conventional wheat is produced in Romania.

On the other hand, other varieties (for example Arieşan) were more contaminated. Arieşan wheat variety is resistant to yellow and brown rust, and medium resistant to *Septoria* contamination and wintering. This variety is characteristic for central and northern Romanian regions, where climatic conditions are characterized by variable humidity and higher rainfall [32]. Thus, the higher contamination of Arieşan variety might be the consequence of a complex dependence on several factors, including agricultural practice and weather parameters.

In the last part of our research, the presence of emerging mycotoxins in wheat-based products for direct human consumption commercialized in Romania was presented. As was also noticed previously by other authors [33], type B ENs were more detected than type A ENs and BEA. The incidences varied from 28% (pasta) to 43% (biscuits) for ENB and from 5% (flour) to 26% (biscuits) for ENB1. The fact that no ENA was detected in the wheat-based products analyzed is interesting, since low levels of ENA were detected in 8% of the wheat samples evaluated. This could be explained by the influence of the cleaning and debranning procedures used during the technological process of wheat that could reduce mycotoxin content [34]. Like in our study, unquantifiable levels of BEA were observed in pasta and biscuits from Italy [35,36]. For ENs, similar incidences have been reported for Italian pasta (4% for ENA, ENA1, and ENB1 and 44% for ENB) [35], and Romanian flour (9%, 11%, 80%, and 17% for ENA, ENA1, ENB, and ENB1, respectively) [37]. Additionally, higher occurrences have been reported in other studies which analyzed Italian pasta (33%, 94%, and 90% for ENA, ENA1, and ENB, respectively) [38], or Spanish refrigerated pizza dough (8% for ENA and 100% for ENA1, ENB, and EB1) [39].

Regarding the concentrations of ENs found in the wheat products, the levels from the present study were higher than those obtained by Quiles et al. [39] in refrigerated pizza dough (maximum of 14.96 μg kg^−1^ for ENA), but were similar to those noted in a study by Juan et al. [35] which reported a maximum concentration of 106 μg kg^−1^ for ENs in pasta. On the other hand, higher levels for ENs were reported by other authors in pasta (maximum of 710 μg kg^−1^ for ENB [38] or 979 μg kg^−1^ for ENB1 [25]), multicereal baby food (maximum of 1100 μg kg^−1^ for ENB) [35], and wheat semolina couscous (maximum of 651 μg kg^−1^ for ENA) [40]. It must be mentioned that, generally, other ingredients than wheat (e.g., oat, maize, rye, rice and some fruits such as nuts, peanuts, raisins) included in wheat-based product recipes could be a source of mycotoxins; in our study, the contribution of these ingredients to mycotoxin content was minimized, as the products analyzed were selected on account of being composed mostly from wheat or white wheat flour.

In the present study, the daily intake of ENs through the consumption of wheat-based products was estimated for the first time for the Romanian population. Furthermore, by including a wide variety of products for direct human consumption commercialized in Romania, such as pasta, flour, breakfast cereals, and biscuits, the estimation was more accurate.

Due to a lack of in vivo toxicity data on BEA and ENs, a TDI or an acute reference dose (ARfD) for BEA or the sum of ENs for humans was not set until now. To obtain some insight into the possible risks of dietary exposure to BEA and the sum of ENs at the estimated levels of exposure, the EFSA Panel on Contaminants in the Food Chain (CONTAM) proposed to compare the estimated chronic exposure levels with the doses reported to cause adverse effects upon therapeutic use of the drug fusafungine via nasal/oromucosal spraying taking a worst case approximation for converting the nasal/oromucosal dose levels to oral dose levels. An oral dose of 90 to 170 μg/kg bw/day was used as a rough estimate for a lowest observed adverse effect level (LOAEL) for a mixture of ENs. In the absence of toxicity data on repeated exposure for BEA, the CONTAM Panel also used this range for BEA. On the other hand, using the Threshold of Toxicological Concern (TTC) approach for human risk assessment (probability of adverse health effects and possible human health risks), the CONTAM Panel found a value of 0.025 μg/kg bw/day for BEA and 1.5 μg/kg bw/day for the sum of ENs [18]. Considering this data and other provisional maximum tolerable daily intakes (PMTDIs) established for various mycotoxins (e.g., 1000 ng/kg bw/day for the sum of DON and its acetylated derivatives [41]), a hypothetic value of 1000 ng/kg bw/day was used for the TDI of the sum of ENs.

Comparing the results obtained in the present study (5.3 ng/kg bw/day for BEA and 80.5 ng/kg bw/day for the sum of ENs for high consumers) with the scenarios proposed by the CONTAM Panel and the hypothetic value proposed in this study, it is easy to observe that the EDIs calculated were lower than all these values. However, the EDI values from the present study were higher than the EDIs of emerging mycotoxins calculated for the Spanish population through the consumption of different wheat-based products. For example, 4.69, 1.08, or 13.19 ng/kg bw/day of ENs could be ingested by the Spanish population as a potential result of high consumption of refrigerated pizza dough [39], bread loaf [42], or pasta [25], respectively. Also, the EDIs of BEA were 0.5 ng/kg bw/day consuming pasta [25] and 4.94 ng/kg bw/day consuming refrigerated pizza dough [39], with both of these values representing an overestimation.

## 4. Conclusions

This study shows that agricultural practices could influence emerging mycotoxin presence in wheat cultivated in Romania. For the four ENs studied, the incidence percentages and mean levels for organic wheat samples were higher than the corresponding values for conventional samples. On a long-time consumption of wheat and wheat-based products, it can be stated that the use of conventional farm practices is safer than organic practices. Wheat-based products for direct human consumption (pasta, flour, breakfast cereals, and biscuits) purchased in Romania were evaluated for emerging mycotoxin occurrence and 32% of them presented detectable levels of BEA, ENA1, ENB, or ENB1. A low EDI of emerging mycotoxins was calculated for the Romanian population (with the maximum being a total of 86 ng/kg bw/day). The approximate risk assessment showed that the total contribution of ENs to the hypothetic TDI (1000 ng/kg bw/day) did not exceed 10%.

Further monitoring studies in different European countries, using sensitive analytical methods, are necessary to confirm the convenience and safety of conventional practices regarding emerging mycotoxin presence in wheat. Also, a multi-variance analysis could be included, as a wide range of compositional, topographic, climatic, and agricultural factors may influence the occurrence and concentrations of mycotoxins in wheat.

## 5. Materials and Methods

### 5.1. Chemical and Reagents

HPLC-grade acetonitrile and methanol were supplied by PanReac AppliChem (Castellar del Vallés, Spain), and LC-MS/MS-grade methanol (≥99.9% purity) was supplied by VWR International Eurolab (Barcelona, Spain). For mobile phases, ammonium acetate (>97%) was supplied by Panreac Quimica S.A.U. (Barcelona, Spain), and acetic acid (100%) was obtained from Merck KGaA (Darmstadt, Germany). Deionized water (<10 MΩ cm^−1^ resistivity) was manufactured in the laboratory using a Milli-Q SP^®^ Reagent Water System (Millipore, Bedford, MA, USA).

Whatman No. 4 filter papers (Maidstone, UK) were used to filter the extract samples. Polypropylene syringes (2 mL) and nylon filters (13 mm diameter, 0.22 μm pore size) were purchased from Análisis Vínicos S.L. (Tomelloso, Spain).

The certified standards of ENs (A, A1, B, and B1) and BEA were purchased from Sigma Aldrich (Madrid, Spain). The individual stock solutions of ENs and BEA were prepared in acetonitrile at 500 μg mL^−1^. Also, a working mixed standard solution in methanol, at concentration of 0.4 μg mL^−1^ for ENs and 0.25 μg mL^−1^ for BEA, was prepared by diluting the individual stock solutions. This solution was used to construct the matrix matched calibration curves. The solutions were stored in glass-stoppered bottles and in darkness under safe conditions at −20 °C.

### 5.2. Sampling

A total of 133 unprocessed wheat samples were collected during the 2014 and 2015 harvest seasons from different Romanian counties in order to investigate the presence of mycotoxins. The samples were divided by agricultural practice as follows: conventional (*n* = 106) and organic (*n* = 27). Information about growing area, type of agriculture, and wheat variety was collected. Wheat samples classified as conventional were divided by variety: Alcantara (*n* = 1), Alex (*n* = 1), Altigo (*n* = 1), Arezzo (*n* = 3), Arieşan (*n* = 7), Balaton (*n* = 2), Boema (*n* = 4), Dropia (*n* = 1), Exotic (*n* = 1), Felix (*n* = 1), Glosa (*n* = 10), Hyfi (*n* = 1), ITC-20 (*n* = 1), Izvor (*n* = 7), Kontrast (*n* = 1), Litera (*n* = 5), Lukulus (*n* = 1), Miranda (*n* = 2), Ponomicus (*n* = 2), Solehio (*n* = 1), Soxenos (*n* = 1), Urbanus (*n* = 1), other types (*n* = 51). The criterion used to include a wheat sample in the study was that wheat must be produced for human consumption. The number of wheat samples for each type (organic or conventional) and each variety was influenced by the frequency of cultivation.

Furthermore, a total of 111 samples of wheat-based products were purchased from different markets located in Cluj-Napoca (Romania) during April to June 2016: white wheat flour (*n* = 41); pasta with minimum of 73% wheat (*n* = 40); breakfast cereals containing between 54% and 90% wheat (*n* = 7); integral biscuits containing between 49% and 95% wheat (*n* = 23). Three wheat-product samples (one biscuit sample and two flour samples) were from organic agriculture. It should be mentioned that in Romania, the organic production and the organic or ecological market offerings are still in the process of development.

Sampling was performed according to the European Union guidelines [43]. Three incremental samples of 1 kg unprocessed wheat were collected in the first seven days after harvesting, obtaining an aggregate sample of 3 kg total weight. In the case of the wheat-based products, two to six packages for each sample were purchased, obtaining an aggregate sample of at least 1 kg total weight. All samples were milled to a fine powder using a laboratory mill. After homogenization, the samples were packed in plastic bags and stored at −20 °C in a dark and dry place until analysis. Three replicates for each sample were weighed for analysis.

### 5.3. Extraction

#### 5.3.1. Wheat

For the wheat extraction, the method of Stanciu et al. [37] was used. Subsamples were weighed (2 g) and placed into 50 mL polytetrafluoroethyl (PTFE) centrifuge tubes, followed by the addition of 10 mL acetonitrile/water (84:16, *v*/*v*). The tubes were stirred for 1 h at 300 shakes min^−1^ using a horizontal shaking device (IKA KS260 basic Stirrer, IKA-Werke GmbH & Co. KG, Staufen, Germany), centrifuged for 5 min at 5 °C and 4500 rpm using an Eppendorf Centrifuge 5810R (Eppendorf, Hamburg, Germany), and then filtered on a Whatman filter paper. Furthermore, 5 mL of supernatant was placed in 15 mL PTFE centrifuge tubes and was evaporated to dryness at 35 °C under a gentle stream of nitrogen using a multi-sample Turbovap LV Evaporator (Zymark, Hopkinton, MA, USA). The residue was reconstituted to a final volume of 1 mL with methanol/water (70:30, *v*/*v*) and filtered through a syringe nylon filter.

#### 5.3.2. Wheat-Based Products

For the wheat-based products, the most suitable extraction procedure was used, according to a previous work [44]. Briefly, subsamples of 2 g, weighed into 50 mL PTFE centrifuge tubes, were extracted with 20 mL of acetonitrile using IKA T18 basic Ultra-Turrax homogenizer (IKA-Werke GmbH & Co. KG, Staufen, Germany) for 3 min. After this, samples were centrifuged for 5 min at 5 °C and 3550 rpm using an Eppendorf Centrifuge 5810R (Eppendorf, Hamburg, Germany) and filtered on a Whatman filter paper. Furthermore, 10 mL of supernatant placed in 15 mL PTFE centrifuge tubes was evaporated to dryness at 35 °C under a gentle stream of nitrogen using a multi-sample Turbovap LV Evaporator (Zymark, Hopkinton, MA, USA). The residue was reconstituted to a final volume of 1 mL with methanol/water (70:30, *v*/*v*) and filtered through a syringe nylon filter.

### 5.4. Mycotoxin Analysis by HPLC-MS/MS

ENs and BEA were analyzed using a LC-MS/MS system, consisting of a LC Agilent 1200 using a binary pump and an automatic injector, and coupled to a 3200 QTRAP^®^ AB SCIEX (Applied Biosystems, Foster City, CA, USA) equipped with a Turbo-V™ source (electrospray ionization) interface. The chromatographic separation was performed at 24 ± 1 °C on a C_18_ reverse phase analytical column Gemini^®^ (3 μm; 150 × 2 mm ID) and a C_18_ guard-column (3 μm; 4 × 2 mm ID) from Phenomenex (Madrid, Spain). Mobile phases were methanol (0.1% acetic acid and 5 mM ammonium acetate) as phase A and water (0.1% acetic acid and 5 mM ammonium acetate) as phase B. The following gradient was used: equilibration at 90% B for 2 min, 80–20 % B in 3 min, 20% B for 1 min, 20–10% B in 2 min, 10% B for 6 min, 10–0% B in 3 min, 100% A for 1 min, 100–50% A in 3 min, return to initial conditions in 2 min and maintain during 2 min. The flow rate was 0.25 mL min^−1^ in all steps. The injection volume was 20 µL.

The QTRAP System was used in the selected reaction monitoring (SRM). The Turbo-V™ source was used in the positive mode with the following settings for Source/Gas Parameters: Vacuum Gauge (10 e-5 Torr) 3.1, curtain gas (CUR) 20, ion spray voltage (IS) 5500, source temperature (TEM) 450 °C, ion source gas 1 (GS1), and ion source gas 2 (GS2) 50. The precursor and product ions and specific parameters used are presented in Table 6. The entrance potential (EP) was the same for all analytes (i.e., 10 V). Acquisition and processing data were performed using Analyst^®^ software, version 1.5.2 (AB SCIEX, Concord, ON, Canada).

The methods of analysis were previously validated [37,44], following the European Union Commission regulations. Matrix-matched calibration curves at concentrations between 1 and 1000 μg kg^−1^ were used to quantify the mycotoxins in the samples analyzed. The LDs and LQs for each mycotoxin and each type of matrix are indicated in Table 5. All experiments were performed in triplicate. Samples presenting levels higher than LDs were considered to estimate the incidence (%) for each mycotoxin.

### 5.5. Statistical Analyses

Statistical analysis was performed using SPSS software (IMB Corp, IBM SPSS Statistics, Armonk, NY, USA), version 22.0 with a statistical significance set at 95% (*p* = 0.05). ANOVA single factor test was used to assess the significance of the differences of the mycotoxin concentrations determined.

### 5.6. Estimation of Daily Intake

The dietary exposure to each mycotoxin was evaluated by calculating the EDI. For this, a deterministic method [45] was applied, using the equation:

(1) $$\text{EDI}\;(\text{ng/kg bw/day})\;=\;\frac{\sum c*K}{N*Bw},$$

where ∑*c* is the sum of each mycotoxin in the samples analyzed (μg kg^−1^), *K* is the daily average consumption per person for the food commodity included in the study (g/capita/day), *N* is the total number of analyzed samples, and *Bw* is the body weight used in the population group.

To obtain the sum of each mycotoxin for the samples analyzed, two different scenarios were designed: one underestimating (lower bound scenario—LB) and another one overestimating (upper bound scenario—UB) the exposure. The LB was obtained by setting a zero value for all samples with levels lower than LQ, whereas the UB was achieved by assigning the LD to those samples with undetected levels and the LQ to those samples with levels between the LD and LQ [46].

To calculate the EDI, the food supply quantity of wheat and products in Romania was considered 369.5 g/capita/day, according to Food and Agriculture Organization of the United Nations Statistics Division (FAOSTAT) [1], and a default average body weight of 70 kg was assumed for Romanian population.

As no TDI for emerging mycotoxins was proposed until now, the risk evaluation was performed taking into account the safety guidelines for other *Fusarium* mycotoxins [19,20]. Consequently, a hypothetic value of 1000 ng/kg bw/day was used for the TDI of the sum of ENs, which is closer or similar to other provisional maximum tolerable daily intakes (PMTDIs) established for various mycotoxins (e.g., 1000 ng/kg bw/day for the sum of DON and its acetylated derivatives [41]). In addition, the same hypothetic TDI value for ENs (1000 ng/kg bw/day) was used previously by other authors [42]. The risk assessment of ENs was carried out by calculating the percentage (%TDI) covered by the EDI from the TDI proposed.

## Figures and Tables

**Table 1:** Summary of enniatin (EN) levels found in Romanian wheat samples distributed by agricultural practice.

Mycotoxin	Parameter	Conventional	Organic	Total
		(*n* = 106)	(*n* = 27)	(*n* = 133)
BEA	Incidence	3	0	3
	LD-LQ	2	0	2
	Frequency (%)	2	0	2
	Mean (μg kg^−1^)	0.07	n.q.	0.07
	Max. (μg kg^−1^)	9.1	n.q.	9.1
ENA	Incidence	6	5	11
	LD-LQ	0	2	2
	Frequency (%)	6	19	8
	Mean (μg kg^−1^)	3.8	7.0	4.5
	Max. (μg kg^−1^)	140	96.6	140
ENA1	Incidence	19	8	27
	LD-LQ	0	0	0
	Frequency (%)	18	30	20
	Mean (μg kg^−1^)	11.2	23.7	13.7
	Max. (μg kg^−1^)	356	272	356
ENB	Incidence	36	19	55
	LD-LQ	0	1	1
	Frequency (%)	34	70	41
	Mean (μg kg^−1^)	42.9	102	54.8
	Max. (μg kg^−1^)	815	487	815
ENB1	Incidence	25	11	36
	LD-LQ	1	0	1
	Frequency (%)	24	41	27
	Mean (μg kg^−1^)	21.5	66.0	30.5
	Max. (μg kg^−1^)	510	510	510

Incidence: number of samples ≥ limit of detection (LD); LD-LQ: number of samples ≥ LD and ≤ limit of quantification (LQ); Frequency: the percentage of samples ≥ LD/total samples; Mean: average of total samples, assuming a zero value for samples ≤ LQ; BEA: beauvericin; n.q.: not quantified because no sample was ≥ LQ.

**Table 2:** Pairs of mycotoxins found in the positive samples of wheat (conventional and organic) and wheat-based products from Romania.

Mycotoxins	Conventional Wheat	Organic Wheat	Wheat Products
	(*n* = 106)	(*n* = 27)	(*n* = 111)
Two mycotoxins			
ENA1 + ENB	1	0	0
ENA1 + ENB1	3	0	0
ENB + ENB1	7	3	16
Three mycotoxins			
ENA1 + ENB + ENB1	9	3	1
BEA + ENB + ENB1	0	0	1
Four mycotoxins			
BEA + ENA1 + ENB + ENB1	1	0	0
ENA + ENA1 + ENB + ENB1	5	5	0

Co-occurrence: number of samples co-contaminated ≥ limit of detection (LD).

**Table 3:** Summary of EN levels found in Romanian conventional wheat samples distributed by cultivars.

Variety	ENA			ENA1			ENB			ENB1			Overall Occurrence
	Occurrence	Mean	Max.	Occurrence	Mean	Max.	Occurrence	Mean	Max.	Occurrence	Mean	Max.	
		(μg kg^−1^)			(μg kg^−1^)			(μg kg^−1^)			(μg kg^−1^)		
Alcantara	0/1	n.q.	n.q.	0/1	n.q.	n.q.	0/1	n.q.	n.q.	0/1	n.q.	n.q.	0/1
Alex	0/1	n.q.	n.q.	0/1	n.q.	n.q.	0/1	n.q.	n.q.	0/1	n.q.	n.q.	0/1
Altigo	0/1	n.q.	n.q.	0/1	n.q.	n.q.	1/1	157.4	157	1/1	12.4	12.4	1/1
Arezzo	0/3	n.q.	n.q.	1/3	3.9	11.8	0/3	n.q.	n.q.	1/3	3.8	11.6	1/3
Arieşan	0/7	n.q.	n.q.	1/7	3.6	25.5	6/7	57.7	149	2/7	15.2	71.1	6/7
Balaton	0/2	n.q.	n.q.	2/2	27.0	43.0	2/2	40.5	65.1	1/2	37.0	74.0	2/2
Boema	0/4	n.q.	n.q.	0/4	n.q.	n.q.	1/4	0.5	2.1	0/4	n.q.	n.q.	1/4
Dropia	0/1	n.q.	n.q.	0/1	n.q.	n.q.	0/1	n.q.	n.q.	0/1	n.q.	n.q.	0/1
Exotic	0/1	n.q.	n.q.	0/1	n.q.	n.q.	1/1	55.7	55.7	1/1	4.5	4.5	1/1
Felix	0/1	n.q.	n.q.	0/1	n.q.	n.q.	0/1	n.q.	n.q.	0/1	n.q.	n.q.	0/1
Glosa	0/10	n.q.	n.q.	1/10	1.8	17.5	3/10	3.9	33.9	1/10	2.0	20.2	3/10
Hyfi	0/1	n.q.	n.q.	0/1	n.q.	n.q.	0/1	n.q.	n.q.	0/1	n.q.	n.q.	0/1
ITC-20	0/1	n.q.	n.q.	0/1	n.q.	n.q.	0/1	n.q.	n.q.	0/1	n.q.	n.q.	0/1
Izvor	0/7	n.q.	n.q.	1/7	9.4	65.6	1/7	22.7	159	1/7	16.6	116	1/7
Kontrast	0/1	n.q.	n.q.	0/1	n.q.	n.q.	1/1	5.2	5.2	0/1	n.q.	n.q.	1/1
Litera	0/5	n.q.	n.q.	1/5	2.8	14.2	1/5	8.0	40.1	1/5	7.4	37.2	1/5
Lukulus	0/1	n.q.	n.q.	0/1	n.q.	n.q.	0/1	n.q.	n.q.	0/1	n.q.	n.q.	0/1
Miranda	0/2	n.q.	n.q.	0/2	n.q.	n.q.	0/2	n.q.	n.q.	0/2	n.q.	n.q.	0/2
Ponomicus	0/2	n.q.	n.q.	0/2	n.q.	n.q.	0/2	n.q.	n.q.	0/2	n.q.	n.q.	0/2
Solehio	0/1	n.q.	n.q.	0/1	n.q.	n.q.	0/1	n.q.	n.q.	0/1	n.q.	n.q.	0/1
Soxenos	0/1	n.q.	n.q.	0/1	n.q.	n.q.	1/1	12.6	12.6	0/1	n.q.	n.q.	1/1
Urbanus	0/1	n.q.	n.q.	0/1	n.q.	n.q.	0/1	n.q.	n.q.	0/1	n.q.	n.q.	0/1
Other types	6/51	7.9	140	12/51	19.5	356	18/51	70.4	815	16/51	37.1	510	23/51

Occurrence: number of samples ≥ limit of detection (LD)/total samples; Mean: average of total samples, assuming a zero value for samples ≤ limit of quantification (LQ); n.q.: not quantified because no sample was ≥ LQ.

**Table 4:** Summary of EN levels found in Romanian wheat-based products.

Mycotoxin	Parameter	Flour	Pasta	Breakfast Cereals	Biscuits	Total
		(*n* = 41)	(*n* = 40)	(*n* = 7)	(*n* = 23)	(*n* = 111)
BEA	Incidence	0	0	0	1	1
	LD-LQ	0	0	0	1	1
	Frequency (%)	0	0	0	4	1
	Mean (μg kg^−1^)	n.q.	n.q.	n.q.	n.q.	n.q.
	Max. (μg kg^−1^)	n.q.	n.q.	n.q.	n.q.	n.q.
ENA	Incidence	0	0	0	0	0
	LD-LQ	0	0	0	0	0
	Frequency (%)	0	0	0	0	0
	Mean (μg kg^−1^)	n.q.	n.q.	n.q.	n.q.	n.q.
	Max. (μg kg^−1^)	n.q.	n.q.	n.q.	n.q.	n.q.
ENA1	Incidence	0	1	0	0	1
	LD-LQ	0	1	0	0	1
	Frequency (%)	0	3	0	0	1
	Mean (μg kg^−1^)	n.q.	n.q.	n.q.	n.q.	n.q.
	Max. (μg kg^−1^)	n.q.	n.q.	n.q.	n.q.	n.q.
ENB	Incidence	12	11	2	10	35
	LD-LQ	1	0	0	3	4
	Frequency (%)	29	28	29	43	32
	Mean (μg kg^−1^)	1.8	10.4	1.9	1.7	4.9
	Max. (μg kg^−1^)	38.2	170	7.8	9.7	170
ENB1	Incidence	2	9	1	6	18
	LD-LQ	0	2	0	3	5
	Frequency (%)	5	23	14	26	16
	Mean (μg kg^−1^)	0.5	1.9	0.5	0.7	1.0
	Max. (μg kg^−1^)	16.6	44.8	3.6	6.2	44.8

Incidence: number of samples ≥ limit of detection (LD); LD-LQ: number of samples ≥ LD and ≤ limit of quantification (LQ); Frequency: the percentage of samples ≥ LD/total samples; Mean: average of total samples, assuming a zero value for samples ≤ LQ; n.q.: not quantified because no sample was ≥ LQ.

**Table 5:** Emerging mycotoxin exposure and risk assessment of the Romanian population through the consumption of wheat-based products.

Mycotoxin	EDI (ng/kg bw/day)		%TDI	
	LB	UB	LB	UB
**BEA**	0	5.3	n.c.	n.c.
**ENA**	0	31.7	0	3.17
**ENA1**	0	10.7	0	1.07
**ENB**	25.8	27.8	2.58	2.78
**ENB1**	5.4	10.3	0.54	1.03
**Sum of ENs**	31.2	80.5	3.12	8.05

EDI: Estimated daily intake; LB: low bound scenario, calculated assuming a zero value for the samples ≤ limit of quantification (LQ); UB: upper bound scenario, calculated assuming the limit of detection (LD) value for the samples ≤ LD and the LQ value for the samples LD-LQ; %TDI: the percentage covered by the EDI from a proposed hypothetic tolerable daily intake (TDI) for the sum of ENs (1000 ng/kg bw/day); n.c.: not calculated because no TDI was proposed for BEA.

**Table 6:** Mass spectrometry (MS/MS) parameters for mycotoxin detection and method sensitivity.

Analyte	Rt	*M*_w_	MS/MS Detection Parameters						Method Sensitivity			
			Precursor Ion	Product Ions	DP	CEP	CE	CXP	Wheat		Products	
									LD	LQ	LD	LQ
	(min)	(g/mol)	(m/z)		(V)				(μg kg^−1^)			
BEA	11.33	783.95	801.2	784.1*^Q^*	116	33	27	10	4	8	1	2
			[M + NH_4_]^+^	244.1*^q^*			39	6				
ENA	13.23	681.90	699.4	210.1*^Q^*	76	30	35	14	6	12	6	12
			[M + NH_4_]^+^	228.2*^q^*			59	16				
ENA1	12.32	667.87	685.4	210.2*^Q^*	66	29	37	8	3	6	2	4
			[M + NH_4_]^+^	214.2*^q^*			59	10				
ENB	10.96	639.82	657.3	196.1*^Q^*	51	28	39	8	1	2	0.5	1
			[M + NH_4_]^+^	214.0*^q^*			59	10				
ENB1	11.57	653.85	671.2	214.1*^Q^*	66	29	61	10	1	2	1	2
			[M + NH_4_]^+^	228.1*^q^*			57	12				

CE: collision energy; CEP: collision cell entrance potential; CXP: collision cell exit potential; DP: declustering potential; LQ: limit of detection; LQ: limit of quantification; Mw: Molecular weight; Rt: Retention time; Q: quantification transition; q: qualification transition.
